# Case Report: Constrictive Pericarditis in a Patient With Isolated Anomalous Right Upper Pulmonary Venous Return

**DOI:** 10.3389/fcvm.2020.612014

**Published:** 2020-12-09

**Authors:** Rody G. Bou Chaaya, Jeremy L. Herrmann, Roopa Akkadka Rao, Mark D. Fisch, Georges Ephrem

**Affiliations:** ^1^Department of Medicine, Indiana University School of Medicine, Indianapolis, IN, United States; ^2^Division of Thoracic and Cardiovascular Surgery, Department of Surgery, Indiana University School of Medicine, Indianapolis, IN, United States; ^3^Krannert Institute of Cardiology, Indiana University School of Medicine, Indianapolis, IN, United States

**Keywords:** constrictive pericarditis, partial anomalous pulmonary venous return (PAPVR), heart failure, warden procedure, cardiac MRI

## Abstract

Thirty-eight-year-old male presented for evaluation of abdominal swelling, lower extremity edema and dyspnea on exertion. Extensive work-up in search of the culprit etiology revealed the presence of an Anomalous Right Upper Pulmonary Venous Return (ARUPVR) into the Superior Vena Cava (SVC). During the attempted repair, the pericardium was found to be thickened and constrictive. Only one other case of co-existent partial anomalous pulmonary venous return and constrictive pericarditis (CP) has been reported. The patient underwent a warden procedure with pericardial stripping with good outcomes at 45 days post-operatively. Thus, the presence of severe heart failure symptoms in the setting of ARUPVR should prompt further investigations. Also, further cases are needed to help guide management in these patients.

## Introduction

Partial anomalous pulmonary venous return (PAPVR) is a rare congenital anomaly that is found in about 0.4–0.7% of autopsies. Once present, patients can be asymptomatic or present with non-specific cardiorespiratory symptoms (1). Constrictive pericarditis is the chronic thickening and scarring of the pericardium resulting in abnormal diastolic filling (2). Co-existence of constrictive pericarditis of nonsurgical etiology and ARUPVR has only been reported once in the literature (3). We herein report the case of a 38 year old gentleman who presented with heart failure symptoms found to have Anomalous Right Upper Pulmonary Venous Return (ARUPVR) and constrictive pericarditis.

## History of Presentation

A 38-year-old gentleman presented to the heart failure clinic for evaluation of abdominal swelling, lower extremity edema, dyspnea on exertion and decreased functional capacity that has progressed over the past 2 years. Recently, he was having recurrent ascites requiring paracentesis. He underwent a GI work up that was negative and was started on diuretics. Physical examination findings were as follows: blood pressure: 121/79 mm Hg, heart rate: 86 beats/min, respiratory rate: 16 breaths/min, temperature: 36.7°C, oxygen saturation of 100% on room air. Jugular venous pressure was elevated. Shifting dullness over the abdomen, and 3+ bilateral lower extremity pitting edema was present. Lungs were clear and no murmur was heard on cardiac auscultation.

## Past Medical History

Medical history is significant for obesity with a BMI of 37, type 2 diabetes mellitus, hypertension, chronic kidney disease stage 3, mild obstructive sleep apnea compliant with CPAP.

## Differential Diagnosis

The differential diagnosis of lower extremity edema, ascites, and dyspnea on exertion in this patient included: right sided heart failure, restrictive cardiomyopathy, pulmonary hypertension, left sided heart failure, constrictive pericarditis, congenital heart disease, and valvular disease. In this setting, an extensive work up was started in search of the culprit etiology.

## Investigations

**Figure 1:**
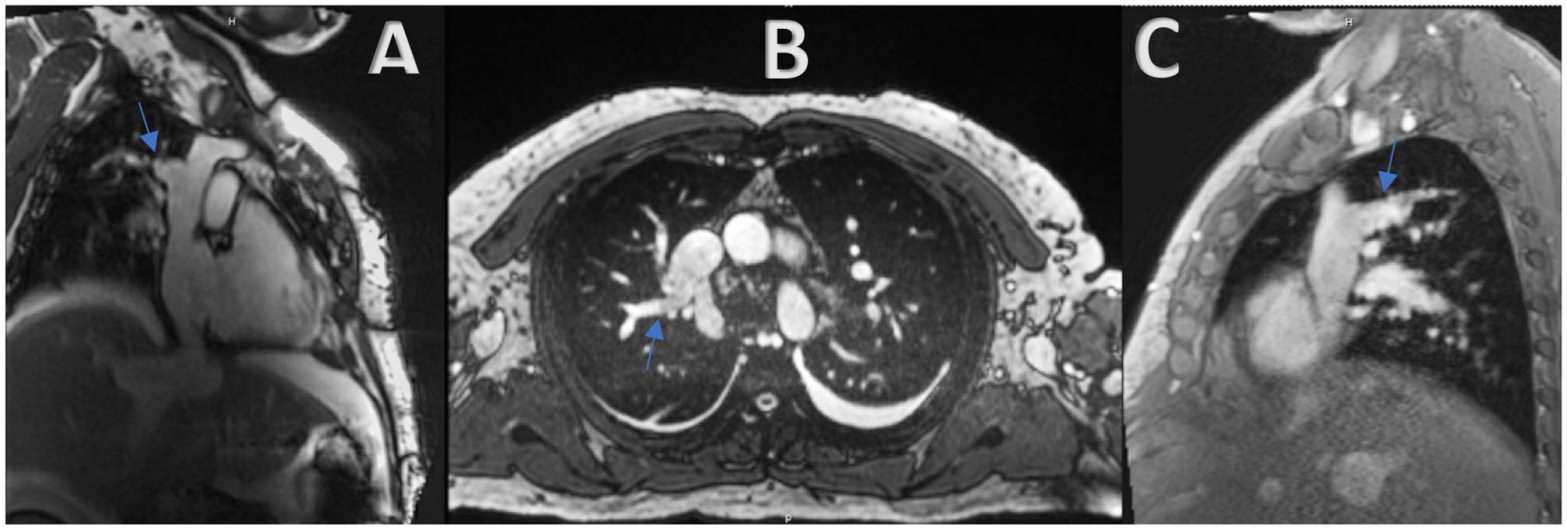
**(A–C)** Cardiac MRI revealing the presence of a large anomalous right upper lobe pulmonary vein draining into the upper SVC (Blue arrow).

**Figure 2:**
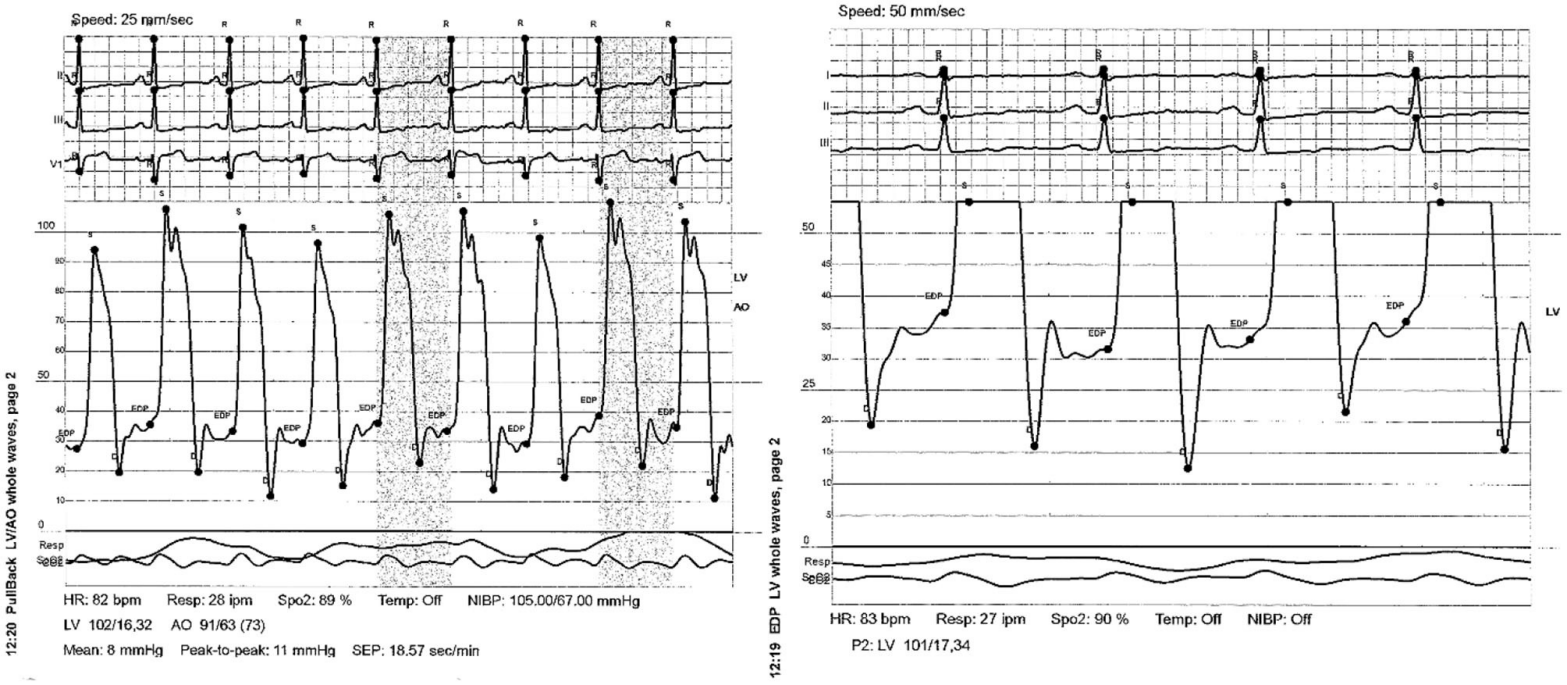
Dip and plateau waveform pattern (square root sign) as seen on the RV pressure tracing.

A 12-lead electrocardiography showed sinus rhythm with low QRS voltage and nonspecific T wave abnormalities. BNP was 110 pg/ml. A transthoracic echocardiography was performed and showed a resting Left Ventricular Ejection Fraction (LVEF) of 50%, normal left atrial and ventricular cavity size, right atrial and ventricular dilatation, elevated RA pressure (>15 mmHg using IVC dynamics), and septal “bounce” without other echocardiographic evidence of constrictive physiology (Supplementary Video 1). No atrial communication was seen. He further underwent right heart catheterization (RHC) that showed equalization of left and right ventricular diastolic pressures (RVDP = LVEDP = 38 mmHg). Pericardial stripping was considered, but an enlarged right ventricle (RV) on echocardiogram elicited further workup by the heart failure team. A cardiac MRI was obtained, and it revealed the presence of a large anomalous right upper lobe pulmonary vein draining into the upper SVC (Figures 1A–C). The estimated QP to QS ratio was 1.35:1. The right ventricle appeared to be moderately to severely enlarged with mildly depressed systolic function (RVEF = 44%; RVEDV: LVEDV = 1.8). The pericardium did not appear to be thickened. Diagnostic right heart catheterization was repeated and showed equalization of diastolic pressures and left to right shunting. Right heart pressures were as follows: RA 31 mmHg; RV 66/33 mmHg; PA 65/36 (47) mmHg; PCWP 32 mmHg. Left ventricular end-diastolic pressure was severely elevated at 32 mmHg. In addition, a dip and plateau waveform pattern (square root sign) was seen on the RV pressure tracing (Figure 2). The directly measured left to right shunt fraction was 1.5–1. RUPV angiography confirmed the presence of the partial anomalous pulmonary venous return (Supplementary Video 2). Left heart catheter yielded normal coronary arteries. Given the previous echocardiographic and right heart catheter findings, constriction was kept on the differential despite absence of thickening on the cardiac MRI. The patient was then referred for PAPVR repair instead of, or in addition to, pericardiectomy.

## Management

**Figure 3:**
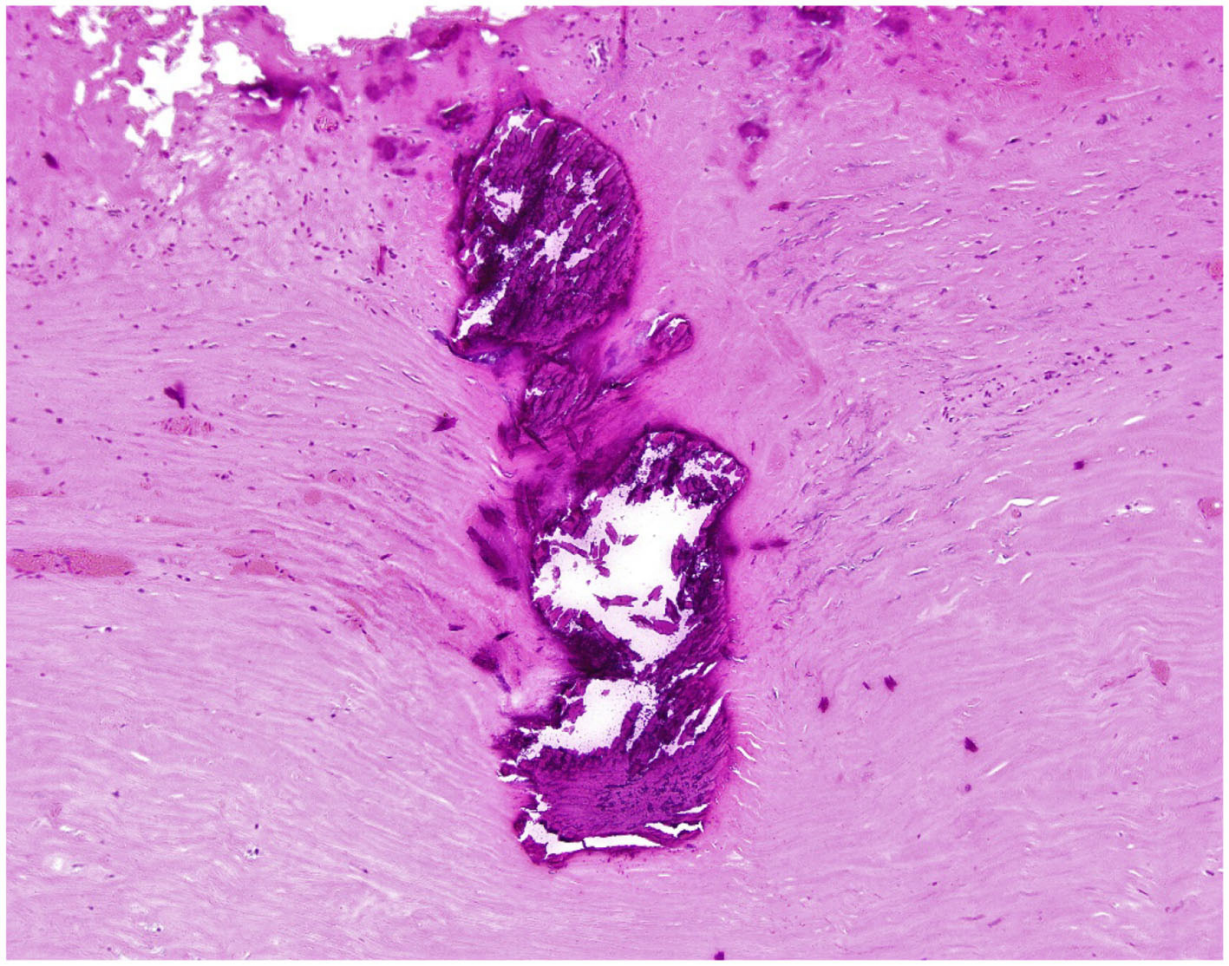
Fragment of pericardium - High-power magnification of the focal calcification and surrounding fibrosis (Hematoxylin & Eosin stain; original magnification 100X).

This case was discussed in our Adult Congenital Heart Disease Multidisciplinary Conference. There was consensus that a surgical repair is recommended to prevent further deterioration of his overall function and heart failure. He was admitted for medical optimization including diuresis and a milrinone infusion. During the operation, the pericardium appeared thickened and constrictive. A subtotal pericardiectomy was performed prior to cardiopulmonary bypass, and 6.5 × 5.2 cm and 5 × 4 cm of pericardial tissue was excised. A Warden procedure was necessary given the high implantation of the ARUPVR. After cardiopulmonary bypass was initiated, and prior to myocardial arrest, the superior vena cava was transected and directly implanted into the right atrial appendage. The heart was then arrested to permit atrial septectomy and construction of an intra-atrial baffle with an expanded polytetrafluoroethylene patch. The patient's postoperative course was uneventful. On pathology, the pericardium showed fibrosis with calcification and was negative for malignancy, acid fast and gram stains (Figure 3).

## Discussion

Partial anomalous pulmonary venous return is a rare congenital anomaly that is found in about 0.4–0.7% of autopsies. Patients with isolated PAPVR tend to be asymptomatic until adulthood (1). This case describes a patient that presented with right heart failure symptoms. Initially, an echocardiogram and a diagnostic RHC suggested the presence of constrictive pericarditis. However, the cardiac MRI showed the presence of PAPVR with no pericardial thickening. This provided a confounding variable that presented a diagnostic dilemma. It is not necessary that the pericardium must be thickened for it to cause CP hemodynamics (4). Nonetheless, an echocardiogram showing a dilated right ventricle goes against CP (2). It is only until the surgery that the concurrence of CP and PAPVR was confirmed. This highlights the importance of early recognition of this combination for appropriate and timely management. Co-existence of constrictive pericarditis of nonsurgical etiology and ARUPVR has only been reported once in the literature (3). In that case, the patient had a similar presentation with dyspnea on exertion, abdominal and bilateral lower extremity swelling. It is unclear if an association exists between these two entities or it is by chance that they co-existed with a combined effect leading to worsening symptoms and causing the patient to present for evaluation. PAPVR is a congenital heart anomaly that comprises <1% of all defects (1). Pericardial defects can be either congenital or acquired, with the congenital variant being exceedingly rare. Fifteen cases of partial or complete absence of the pericardium that were found over 40 years among 34,000 patients undergoing cardiovascular surgery (0.044%), none of which were diagnosed preoperatively. Pericardial defects have been described in patients with congenital heart conditions. But the associations have been broad and infrequent making it unlikely that most of these defects are related to the pathogenic mechanisms of other congenital heart diseases (5). In comparison to pericardial defects, congenital pericardial thickening has been reported in association with congenital heart defects in a very limited setting. This includes congenital connective tissue diseases such as Myhre Syndrome and rare mutations causing insufficient lubrication of the pericardial and synovial fluid such as camptodactyly-arthropathy-coxa vara-pericarditis (CACP) syndrome (6, 7). The only other time this combination was explored was in the case report previously mentioned. This makes the possibility of a connection or a causal relation between constrictive pericarditis and ARUPVR unlikely. In our case, the patient had no history of thoracic surgery, radiation, connective tissue disorder, trauma, or uremia. Also, the pericardium pathology was negative for malignancy, acid fast and gram stains. The etiology of CP in our patient could have been a slow ongoing process of unknown etiology (viral etc.) that exacerbated a preexisting PAPVR. Current guidelines recommend surgical repair for PAPVR when the pulmonary to systemic flow is >1.5:1 (8). Surgical repair may include internal baffling if the anomalous PV enters near the SVC-RA junction or a Warden procedure if the anomalous PV enters the SVC more proximally (i.e., above the right pulmonary artery) (9). Pericardiectomy remains the mainstay of treatment for chronic constrictive pericarditis (10). No guidelines exist regarding the best approach in management of patients with constrictive pericarditis and co-existent PAPVR. Recent limited literature has demonstrated no immediate post-operative complications in those that underwent pericardiectomy with concomitant warden procedure (3). Our patient's post-operative course was uneventful with good outcomes at 10 days, and 1 month follow up.

## Follow Up

The patient was seen in advanced heart failure clinic 10 days after procedure for follow up. His dyspnea of exertion, lower extremity swelling, and ascites were greatly improved. At 1-month post-op, he presented to the cardiothoracic surgery clinic. His symptoms further improved, his weight remained 13 lbs. lower than his discharge weight and he did not require any cardiovascular rehospitalizations. Repeat echocardiogram done at 45 days post-op showed normal LV and RV systolic and diastolic function, along with mild right atrial and RV dilation.

## Conclusion

The combination of ARUPVR and CP is a rare occurrence. It can be difficult to diagnose and may be overlooked until older age. When ARUPVR is diagnosed, the presence of severe heart failure symptoms should prompt further investigations as patients are mostly asymptomatic in its pure form. Early recognition of this combination leads to appropriate and timely management. A Warden procedure with pericardial stripping may alleviate right heart overload with an acceptable risk profile. Further cases are needed to help guide management in these patients.

## Learning Objectives

1. The presence of right heart dilatation prompts scrutiny of the pulmonary venous anatomy.
2. Severe heart failure symptoms in the setting of ARUPVR warrants further investigations to rule out additional culprits.
3. To highlight a rare combination of ARUPVR and non-surgical CP.
4. A warden procedure with pericardial stripping seems to offer good outcomes in patients presenting with ARUPVR and CP.

## Data Availability Statement

The raw data supporting the conclusions of this article will be made available by the authors, without undue reservation.

## Ethics Statement

Ethical review and approval was not required for the study on human participants in accordance with the local legislation and institutional requirements. The patients/participants provided their written informed consent to participate in this study.

## Author Contributions

RB took the lead in writing the manuscript. All authors cared for the patient, provided critical feedback, and helped shape and edit the final manuscript.

## Conflict of Interest

The authors declare that the research was conducted in the absence of any commercial or financial relationships that could be construed as a potential conflict of interest.

## Supplementary Material

Supplementary video 1 Echocardiogram with evidence of septal bounce compatible with constrictive physiology.

Supplementary Video 2 RUPV angiography confirming the presence of the partial anomalous pulmonary venous return.
