# Supplementary material for: Case Report: Constrictive Pericarditis in a Patient With Isolated Anomalous Right Upper Pulmonary Venous Return
Source: Front Cardiovasc Med. 2020 Dec 9;7:612014. doi: 10.3389/fcvm.2020.612014 (PMC7768637; doi:10.3389/fcvm.2020.612014)
Supplement: Supplementary file 3 [file Table_1.DOCX]

| At presentation | - Abdominal swelling, lower extremity edema, dyspnea on exertion and decreased functional capacity of 2 years duration - EKG showing sinus rhythm with low QRS voltage and nonspecific T wave abnormalities - TTE demonstrating an EF of 50%, right atrial and ventricular dilation, and “septal bounce” - Normal laboratory workup including BNP - Increased diuretics dose and continued paracentesis as needed |
| --- | --- |
| 1 week after presentation  10 days after presentation  3 weeks after presentation  4 weeks after presentation  10 weeks after presentation | - Right heart catheterization showing equalization of left and right ventricular diastolic heart pressures - Cardiothoracic surgery appointment for consideration of pericardial stripping - Discussion with heart failure team to obtain cardiac MRI as right heart was dilated on TTE. - Cardiac MRI revealed the presence of a large anomalous right upper lobe pulmonary vein draining into the upper SVC - right heart catheterization was repeated and showed equalization of diastolic pressures and left to right shunting with a dip and plateau waveform pattern - Adult congenital heart disease multidisciplinary conference with decision to proceed with surgery - Surgery day: pericardium was found to be constrictive, pericardiectomy was performed followed by a warden procedure - Post-operative course uneventful - Started on lisinopril and metoprolol as BP improved. Continued with torsemide. - Follow up appointment: dyspnea of exertion, lower extremity swelling, and ascites were greatly improved - Torsemide dose decreased, lisinopril and metoprolol dose increased - Repeat TTE showing normal LV and RV systolic and diastolic function, along with mild right atrial and RV dilation |
| *EKG: electrocardiogram; TTE: trans thoracic echocardiogram; EF: ejection fraction; BNP: B-type natriuretic peptide; MRI: magnetic resonance imaging; SVC: superior vena cava; BP: blood pressure; LV: left ventricle; RV: right ventricle* | |
